# Diversity and Geographic Distribution of Ligninolytic Fungi Associated With *Castanopsis sieboldii* Leaf Litter in Japan

**DOI:** 10.3389/fmicb.2020.595427

**Published:** 2020-11-23

**Authors:** Takashi Osono, Shunsuke Matsuoka, Dai Hirose

**Affiliations:** ^1^Faculty of Science and Engineering, Doshisha University, Kyoto, Japan; ^2^Graduate School of Simulation Studies, University of Hyogo, Kobe, Japan; ^3^School of Pharmacy, Nihon University, Funabashi, Japan

**Keywords:** biogeography, bleaching, fungal community, spatial structure, decomposition

## Abstract

The diversity and geographic pattern of ligninolytic fungi were investigated within the distribution range of an evergreen tree, *Castanopsis sieboldii* (Fagaceae), in Japan. Fungal isolates obtained from 18 sites in subtropical and temperate regions in Japan were classified into 50 operational taxonomic units in Ascomycota and Basidiomycota according to the base sequence of the rDNA internal transcribed spacer region. Ordination by nonmetric multidimensional scaling showed the separation of fungal compositions between the study sites which was significantly related to the latitude, longitude, and mean annual temperature (MAT) of the study sites. We applied variation partitioning to separate the magnitude of the climatic, spatial, and leaf property factors and found the roles of MAT and spatial factors in structuring fungal assemblages, suggesting the importance of both niche processes and such non-niche processes as priority effect and dispersal limitation. The bleached area on leaf litter was greater at sites with higher MAT and precipitation located at lower latitudes and at sites where some major ligninolytic fungi occurred at greater relative frequencies, indicating that not only the climatic conditions but also the biogeographic patterns of distribution of ligninolytic fungi influence the decomposition of lignin in leaf litter.

## Introduction

Fungi play central roles in the decomposition of lignin and other recalcitrant compounds, such as cutin and tannin, in leaf litter of forest trees (van der Wal et al., 2013; Baldrian, 2017). Fungi that selectively remove lignin cause extensive whitening (denoted here as bleaching) of litter materials and stimulate the turnover of carbon and nutrients in soil (Osono, 2020). Previous studies have demonstrated that fungi on leaf litter, including ligninolytic ones associated with the bleaching, show biogeographical patterns and respond sensitively to environmental change along climatic and elevational gradients at population and community levels (van Maanen et al., 2000; Gourbière et al., 2001; Tokumasu, 2001; Hagiwara et al., 2015; Matsukura et al., 2017). Such biogeographic patterns of fungal diversity and composition have been reported to be influenced by climatic, edaphic, and vegetation factors, of which the climate is of particular importance (Peay et al., 2016; Bahram et al., 2018; Větrovský et al., 2019). In addition to the climate, recent studies have demonstrated the importance of spatial factors, such as priority effect and dispersal limitation, in producing the biogeographic patterns of fungal community composition across a range of spatial and temporal scales (Tedersoo et al., 2014; Peay et al., 2016), which contrasts to early predictions that fungi are probably long-distance dispersers and that their propagules are everywhere (Wit and Bouvier, 2006).

Considering the magnitude of ongoing global environmental change, it is crucial to explore how climate, vegetation, spatial, and other factors interact to affect the patterns of diversity and biogeography of fungi associated with leaf litter decomposition. However, factors affecting the biogeography of bleaching fungi on leaf litter have rarely been explored. In Asian forests of different climatic regions, Osono (2011) demonstrated that the diversity and species composition of fungal assemblages on leaf litter were closely related to the climatic gradient. In that study, however, the host tree species examined for fungi were different between the climatic regions, making it difficult to separate the effect on the fungal assemblages of climatic conditions from that of host tree species. Studies are thus needed on geographical patterns of bleaching fungi on leaf litter of single host tree species that are distributed along climatic gradients. We hypothesize that the taxonomic richness and composition of bleaching fungi associated with leaf litter of a single host tree species showed a geographic pattern and were related to climatic conditions, such as mean annual temperature (MAT), as well as spatial factors.

**FIGURE 1:**
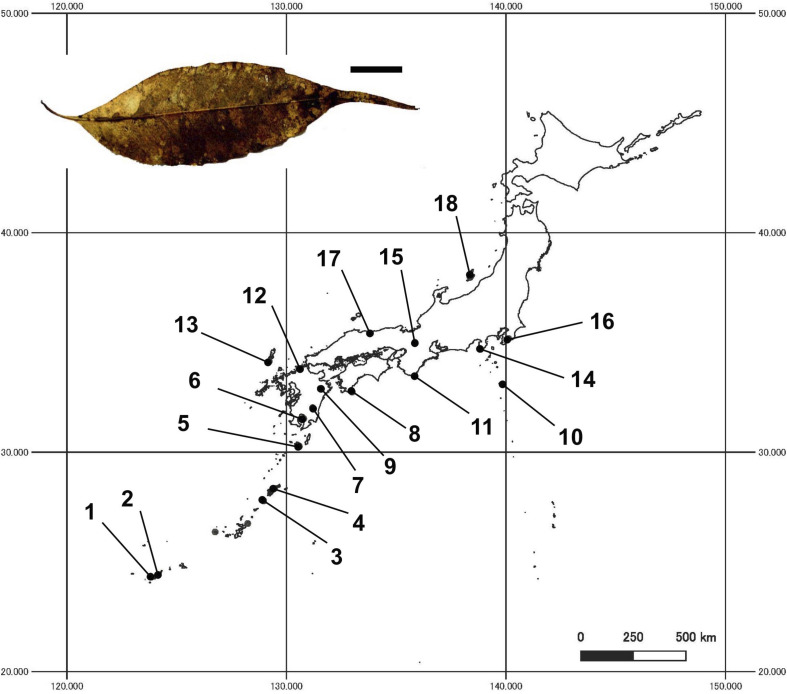
Locations of 18 study sites in Japan, and a photo of a bleached leaf litter (bar = 1 cm). Site numbers are as in Table 1.

The purposes of the present study were to examine (i) the taxonomic richness and composition of ligninolytic fungi associated with the bleaching of leaf litter and (ii) the magnitude of relative importance of climate, leaf quality, and spatial factors affecting the composition of fungal assemblages within the distributional range of a single host tree species *Castanopsis sieboldii* (Makino) Hatus. ex T.Yamaz. et Mashiba (Fagaceae) in Japan. We chose *C. sieboldii* as the focal host species because this species is widely distributed across different climate regions of the Japanese archipelago (Aoki et al., 2014). Eighteen study sites in subtropical and temperate regions that cover almost the entire range of natural distribution of *C. sieboldii* in Japan were selected for the present study. This tree species is a dominant, canopy, and climax species in Japanese *Castanopsis*-type evergreen broad-leaved forests. These *Castanopsis*-type forests have features characteristic of the biodiversity and endemism of the subtropical and warm-temperate zones in Japan. The focal tree species is evergreen broad-leaved and relatively shade-tolerant. Bleached portions are obviously and easily found on the surface of these forests’ leaf litter (Osono et al., 2008; Figure 1), making this leaf litter a suitable material for the study of the biogeographic pattern of ligninolytic fungi associated with the bleaching. Finally, bleached leaf area was measured at the study site so as to examine the roles of climatic factors, leaf property, and fungal occurrence on the geographic patterns of emergence of fungal bleaching on leaf litter.

## Materials and Methods

### Study Sites and Sampling

Samples were collected at 18 study sites in subtropical and temperate regions in Japan (24.307 to 37.966°N, 123.847 to 140.120°E, 45 to 440 m a.s.l.; Table 1 and Figure 1). The study sites were located in mature evergreen broad-leaved forests dominated by *C. sieboldii*, and cover almost the entire range of natural distribution of *C. sieboldii* in Japan, from Iriomote Island of the Ryukyu Islands, adjacent to the present southern boundary of Japan, to Sado Island, adjacent to its northern range. We selected the study sites based on following criteria although details in histories of the individual site are unknown: (i) forest canopy is closed and forest contains at least 20 individuals of *C. sieboldii* with diameter at breast height (DBH) >20 cm and (ii) there is no sign of large-scale natural and anthropogenic disturbance. Latitudes and longitudes of the sites are strongly correlated (Pearson’s *r* = 0.820, *n* = 18, and *P* < 0.001), as the Japanese Archipelago is elongated from northeast to southwest.

**TABLE 1:** Location, climates, sampling month, bleached area on leaf litter (% total leaf area), leaf properties, and the number of fungal isolates for 18 study sites.

Site No.	Site name	Latitude (°N)	Longitude (°E)	Elevation (m)	MAT (°C)	MAP (mm)	Maximum snow depth (cm)	Sampling month	Bleached area	LMA (mg/cm^2^)	AUR content (mg/g)	Number of isolates
1	Iriomote Is., Okinawa	24.307	123.847	176	23.2	2738.9	0	Sept 07	36.7 (4.9)	8.0 (0.7)	289	13
2	Ishigaki Is., Okinawa	24.419	124.167	72	23.0	2352.1	0	Sept 07	36.3 (4.5)	8.5 (0.5)	290	14
3	Tokunoshima Is., Kagoshima	27.774	128.964	286	19.7	2386.8	0	Sept 08	24.0 (3.1)	8.5 (0.3)	269	9
4	Amami-Oshima Is., Kagoshima	28.338	129.446	326	19.1	2897.1	0	Sept 08	31.8 (2.2)	8.3 (0.2)	284	6
5	Yakushima Is., Kagoshima	30.257	130.585	195	19.1	3589.8	0	Aug 09	26.3 (5.3)	9.2 (0.2)	306	7
6	Mt. Takakuma, Kagoshima	31.525	130.787	440	14.8	3014.8	0	Sept 08	12.7 (2.3)	8.0 (0.3)	312	10
7	Aya, Miyazaki	32.027	131.194	241	15.4	2838.8	0	Sept 09	18.0 (1.4)	10.2 (0.7)	301	5
8	Cape Ashizuri, Kochi	32.740	132.999	271	16.9	2458.8	0	Jul 10	19.6 (2.7)	8.3 (0.3)	304	21
9	Bungo-Ohno, Oita	32.894	131.540	289	14.3	2350.9	0	Sept 09	23.6 (2.6)	7.7 (0.3)	303	2
10	Hachijo Is., Tokyo	33.108	139.841	333	16.8	3014.6	0	Aug 12	22.5 (3.3)	7.8 (0.2)	318	23
11	Kii-Oshima, Wakayama	33.466	135.833	94	17.0	2547.1	0	Aug 11	14.5 (3.3)	8.2 (0.3)	288	15
12	Shiroyama, Fukuoka	33.815	130.590	113	15.5	1691.7	0	Sept 09	15.0 (1.6)	8.6 (0.4)	280	10
13	Tsushima Is., Nagasaki	34.142	129.218	347	14.0	2122.7	0	Sept 09	14.0 (3.0)	9.6 (0.5)	278	13
14	Izu Peninsula, Shizuoka	34.691	138.839	135	14.9	2042.9	2	Aug 11	21.1 (1.8)	8.2 (0.4)	265	13
15	Otsu, Shiga	35.004	135.857	130	14.3	1496.2	0	Jul 10	5.9 (1.5)	7.5 (0.7)	294	4
16	Kiyosumi, Chiba	35.176	140.120	195	13.5	2075.7	5	Aug 11	8.2 (2.2)	9.8 (0.6)	284	10
17	Kurayoshi, Tottori	35.425	133.824	45	14.2	1921.9	25	Sept 12	10.8 (1.5)	9.3 (0.5)	259	7
18	Sado Is., Niigata	37.966	138.368	73	12.9	1838.9	21	Sept 13	11.5 (1.2)	8.1 (0.3)	285	3

*Values are means with standard errors in parentheses (*n* = 10). MAT, mean annual temperature; MAP, mean annual precipitation; LMA, leaf mass per area; AUR, acid unhydrolyzable residue. The site numbers are arranged in the order of latitude.*

Sampling was conducted once at each site in the summer (from July to September) from 2007 to 2013. At each site, we randomly set 10 quadrats (15 × 15 cm) at least 3 m from each other. A total of 20 leaves of *C. sieboldii* with evident bleaching and of which more than half of the original leaf area remained were collected from the surface of the forest floor within the quadrats, two leaves per quadrat. These leaves were placed in paper bags and preserved at 4°C for no longer than 3 days before the isolation of fungi.

Additional leaves of *C. sieboldii* with more than half of their original leaf area remaining were collected from the surface of the forest floor within the quadrat and were used to measure the bleached leaf area and total leaf area to calculate the relative bleached area according to the method described in Hagiwara et al. (2015). The bleached area is expressed as a percentage of the total leaf area, and the mean value of the bleached area was calculated for each study site (Table 1). Leaf disks (5.5 mm in diameter) were then excised from the bleached area and used for measurements of leaf mass per area (LMA) and content of acid unhydrolyzable residues (AUR) according to the methods described in Osono et al. (2008). The AUR fraction contains a mixture of organic compounds in various proportions, including condensed tannins, phenolic and carboxylic compounds, alkyl compounds such as cutin, and true lignin (Preston et al., 1997).

### Fungal Isolation

Single leaf disks were excised from the bleached portion of individual leaves, using a sterile 5.5-mm-diameter cork borer, produced 20 leaf disks of bleached portions from each study site. Fungi were isolated from the disks using the surface disinfection method described by Osono et al. (2008). The surface-disinfected materials were plated on 9-cm Petri dishes containing lignocellulose agar (LCA) modified as described by Miura and Kudo (1970), two disks per plate. The plates were incubated at 20°C in darkness and observed for 4 weeks after the plating. Any fungal hyphae or spores that appeared on the plates were transferred to fresh LCA plates, incubated, and observed micromorphologically.

Isolates were tested for their potential activity to bleach leaf litter under a pure culture condition. Newly shed leaves of *C. sieboldii* without obvious fungal or faunal attack were oven-dried at 40°C for 1 week. The leaves were cut into pieces (approximately 2 cm × 2 cm) and autoclaved at 120°C for 20 min. The sterilized leaf pieces were placed on the surface of Petri dishes (6 cm diameter), one piece per plate, containing 20 ml of 1% malt extract agar [malt extract 2% and agar 2% (w/v)] previously inoculated with fungal isolates and incubated at 20°C for 12 weeks in the dark. After incubation, the leaf pieces were examined under a binocular microscope with magnification of 40× for the occurrence of bleaching on the leaf surface. Fungal isolates that caused bleaching were regarded as ligninolytic fungi. We judged 185 fungal isolates from 360 leaf disks (18 study sites × 20 leaves) to be ligninolytic fungi (Supplementary Table 1) and used them for molecular analysis as described below. Three control plates with sterilized leaves but no fungal inoculation were also incubated under the same conditions. No microbial colonies or bleaching developed on the control plates, indicating the effectiveness of the sterilization method used.

### Determination of OTUs

The DNA sequences of the 185 samples of fungal isolates were analyzed. DNA extractions and PCR and sequencing reactions followed similar methods to those described by Hirose et al. (2016). The rDNA internal transcribed spacer (ITS) region was amplified using primers ITS1f (Gardes and Bruns, 1993)/LR3 (Vilgalys and Hester, 1990). The ITS sequences determined were compared with the rDNA sequences available in the GenBank database by means of BLAST+ (Camacho et al., 2009) and assigned taxonomically. The sequences determined in the present study were deposited in the DNA Data Bank of Japan (DDBJ; LC505073-LC505341; Supplementary Table 1).

The procedures used for bioinformatics analyses followed those described in Matsuoka et al. (2016). First, the whole rDNA ITS (i.e., ITS1-5.8S-ITS2) region of each sequence was extracted using ITSx version 1.0.11 (Bengtsson-Palme et al., 2013). The rDNA ITS sequences were then assembled using Claident pipeline v0.1.2013.08.10 (software available online: https://www.claident.org/). All sequences were assembled across the samples preliminarily at a threshold similarity from 90 to 99% at a 1% interval, and we adopted the threshold similarity of 96% in the present study because the number of operational taxonomic units (OTUs) increased abruptly at 97% sequence similarity. The resulting consensus sequences at 96% sequence similarity represented molecular OTUs. Record was kept of one fungal isolate in the present study even when more than two isolates belonging to the same OTU were obtained from single leaf disk. For each of the OTUs, taxonomic identification was conducted based on the query-centric auto-k-nearest-neighbor (QCauto) method (Tanabe and Toju, 2013) with the reference database from International Nucleotide Sequence Database Collaboration (INSDC) and subsequent taxonomic assignment with the lowest common ancestor (LCA) algorithm (Huson et al., 2007) as implemented in Claident. A benchmark analysis has shown that the combination of the QCauto and LCA algorithms is less susceptible to erroneous sequences in database and returns the accurate taxonomic identification results among the existing methods of automated DNA metabarcoding (Tanabe and Toju, 2013). The results of Claident are given in Supplementary Table 2. Those OTUs with more than one isolates were regarded as major OTUs. Additional Blast search was conducted for taxonomic identification of the major OTUs.

### Data Analyses

We prepared a datasheet of fungal assemblages indicating the number of isolates in each fungal OTU occurred for each site and calculated relative frequency of each OTU at each site as a percentage of the number of isolates with respect to the total number of isolates (i.e., 185). Data of air temperature, precipitation, and maximum snow depth were obtained from the nearest station of the Automatic Metrological Data Acquisition System (Japan Meteorological Agency) to each study site. We used nonmetric multidimensional scaling (NMDS) with the Bray-Curtis distance metric to analyze the variation of composition of fungal assemblages. The NMDS analysis was carried out with the metaNDS function with default settings of the vegan package of R software (Oksanen et al., 2019). The envfit command of the vegan package was then used to examine correlation of NMDS structure with the sampling month (July, August, and September), latitude, longitude, elevation, MAT, mean annual precipitation (MAP), maximum snow depth, LMA, and AUR content, by permutation tests (9999 permutations). One-way permutational multivariate analysis of variance (PERMANOVA) was conducted to explore whether the dissimilarity of OTU composition was related to the sampling month, geographical coordinates, climatic variables, and leaf properties.

We conducted the variation partitioning with distance-based redundancy analysis (db-RDA) to quantify the contribution of the environmental, spatial, and leaf property variables to the fungal assemblages (Peres-Neto et al., 2006). The detailed procedures are described in Matsuoka et al. (2016). First, data of fungal assemblages for 18 study sites were converted into the Bray-Curtis dissimilarity matrix. Then, we constructed environmental, spatial, and leaf property models. In the environmental model, forward selection procedure with 999 permutations with an alpha criterion = 0.05 (Blanchet et al., 2008) was performed for four environmental variables (mean annual temperature, annual precipitation, maximum snow depth, and elevation). In the spatial model, spatial variables extracted based on Moran’s Eigenvector Maps (MEM; Borcard et al., 2004) using the dbmem command in the R adespatial package. The forward selection procedure was then performed for the MEM vectors to select spatial variables that significantly influenced community dissimilarities. In the leaf property model, forward selection procedure with 999 permutations with an alpha criterion = 0.05 was performed for two variables (LMA and AUR). Based on these three models, adjusted *R*^2^ values for the environmental fraction, the spatial fraction, the leaf property fraction, and their shared fraction (Peres-Neto et al., 2006) were calculated using the capscale command in the vegan package.

We used generalized linear models (GLMs) to examine singly the effects of sampling month, geographical coordinates, climatic variables, leaf properties, and relative frequencies of major fungal OTUs on the bleached leaf area. An automatic stepwise model selection with Akaike information criterion (AIC) was performed to find the most parsimonious model, using the minimum AIC as the best-fit estimator. The error structure of the GLM was Gaussian. The glm function in the R software was used to perform the analysis.

## Results

### Taxonomic Composition

A total of 185 fungal isolates possessing ligninolytic activity were obtained from 360 leaf disks from bleached portions of leaf litter collected at 18 sites (Supplementary Table 1), two to 23 isolates per site (Table 1). These fungi were classified into 50 OTUs, with 25 OTUs belonging each to Ascomycota (116 isolates) and to Basidiomycota (69 isolates; Supplementary Table 2). Of the 50 OTUs, 18 OTUs consisted of more than one isolate and regarded as major OTUs (Table 2), while the other 32 OTUs were singletons. The most frequent OTU was *Nemania diffusa* OTU_01, accounting for 25% (46) of the 185 isolates, followed by *Nemania bipapillata* OTU_02, *Gymnopus* sp. OTU_03, and *Nodulisporium* sp. OTU_04 (Table 2). All 25 OTUs in the Ascomycota belonged to Xylariaceae (Sordariomycetes) in such genera as *Xylaria, Hypoxylon, Nemania, Astrocystis, Biscogniauxia*, and *Nodulisporium* (Supplementary Table 2). The 25 OTUs in the Basidiomycota belonged to Agaricales or Russulales (Agaricomycetes); OTUs in Agaricales were assigned to *Mycena* in Tricholomataceae and *Gymnopus, Marasmius, Marasmiellus*, and *Crinipellis* in Marasmiaceae, and OTUs in Russulales belonged to Lachnocladiaceae (Supplementary Table 2). Six OTUs in the Ascomycota were assigned successfully to species by Claident, whereas no OTUs in the Basidiomycota were identified to species (Supplementary Table 2).

**TABLE 2:** Taxon, number of isolates and sites of 18 major fungal OTUs.

OTU	Taxon	Phylum	Number of isolates	Number of sites
OTU_01	*Nemania diffusa*	Ascomycota	46	13
OTU_02	*Nemania bipapillata*	Ascomycota	20	3
OTU_03	*Gymnopus* sp.	Basidiomycota	20	8
OTU_04	*Nodulisporium* sp.	Ascomycota	11	5
OTU_06	Agaricomycetes sp.	Basidiomycota	10	7
OTU_05	*Xylaria arbuscula*	Ascomycota	9	6
OTU_07	*Xylaria cubensis*	Ascomycota	6	4
OTU_08	Agaricomycetes sp.	Basidiomycota	5	3
OTU_09	*Gymnopus* sp.	Basidiomycota	5	4
OTU_10	Agaricales sp.	Basidiomycota	4	4
OTU_12	Russulales sp.	Basidiomycota	3	2
OTU_14	*Xylaria cubensis*	Ascomycota	2	1
OTU_15	*Gymnopus* sp.	Basidiomycota	2	1
OTU_16	*Crinipellis* sp.	Basidiomycota	2	2
OTU_18	Lachnocladiaceae sp.	Basidiomycota	2	2
OTU_19	*Hypoxylon monticulosum*	Ascomycota	2	1
OTU_20	*Xylaria* sp.	Ascomycota	2	1
OTU_21	Xylariaceae sp.	Ascomycota	2	1

### Geographic Pattern

**FIGURE 2:**
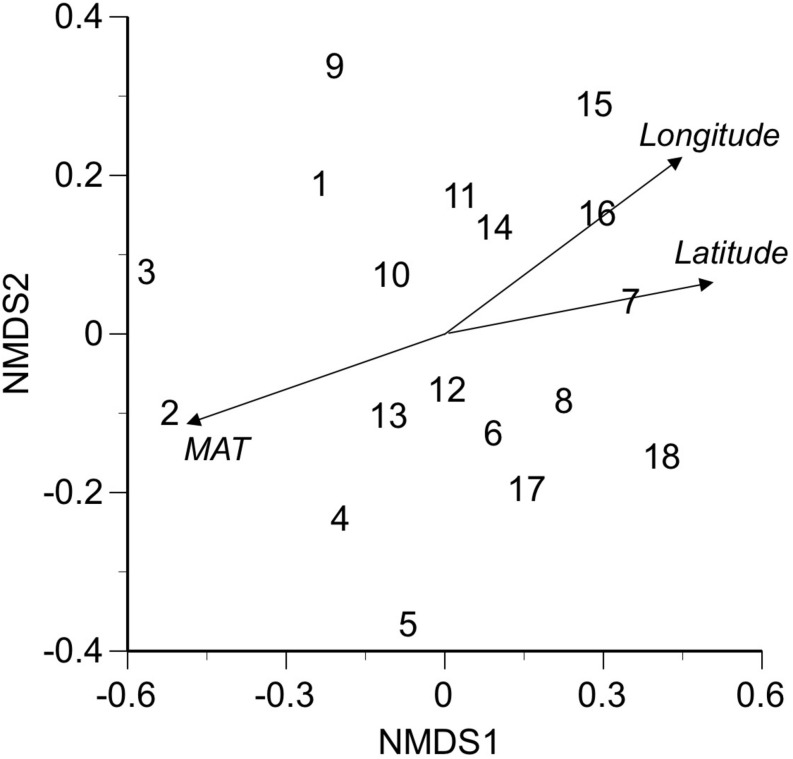
Multivariate ordination of fungal assemblages in bleached portions of leaf litter at 18 sites, using nonmetric multidimensional scaling (NMDS, stress value = 0.154). Site numbers and abbreviation are as in Table 1. Three variables (latitude, longitude, MAT; italicized) with significant effects are indicated by arrows.

**FIGURE 3:**
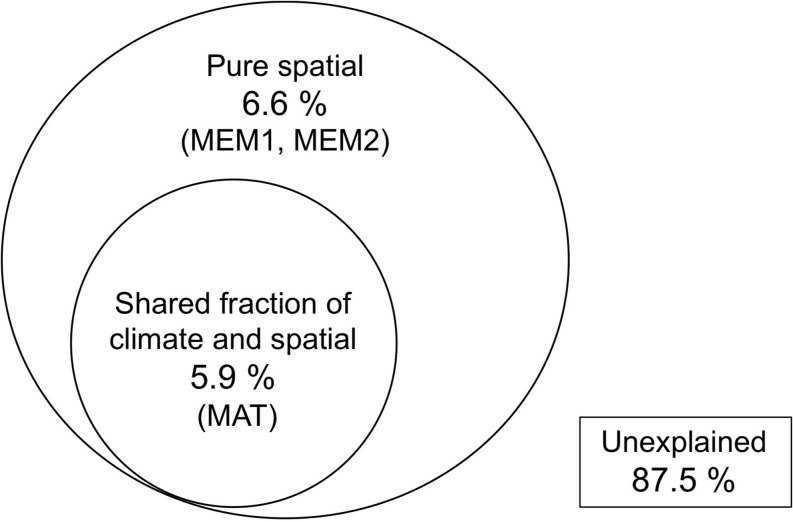
Venn diagram showing pure and shared effects of spatial distance and climate on fungal assemblage composition as derived from variation partitioning analysis. Selected variables are in parentheses. No variables were selected in the leaf property model, i.e. the proportion of explained variation was zero.

*Nemania diffusa* OTU_01 occurred 13 of the 18 sites, followed by *Gymnopus* sp. OTU_03 (8 sites), Agaricomycetes sp. OTU_06 (7 sites), and *Xylaria arbuscula* OTU_05 (6 sites; Table 2). The NMDS ordination showed the separation of fungal OTU compositions between the study sites (Figure 2; stress value = 0.154). The ordination was significantly correlated with the latitude (*R*^2^ = 0.610, *P* = 0.0008), longitude (*R*^2^ = 0.473, *P* = 0.0067), and MAT (*R*^2^ = 0.556, *P* = 0.002; Figure 2), but was not significantly (*P* > 0.05) correlated with the sampling month, elevation, MAP, maximum snow depth, LMA, or AUR content (“envfit” function). In the PERMANOVA, the fungal OTU composition was significantly affected by latitude (*F*-value = 1.52, *R*^2^ = 0.087, and *P* = 0.039) and longitude (*F*-value = 1.73, *R*^2^ = 0.099, and *P* = 0.011). In the variation partitioning (Figure 3), MAT was selected as an environmental variable, and two MEM vectors (MEM1 and MEM2) were selected as spatial variables. Values of MEM1 were significantly correlated with the longitude (Pearson’s *r* = 0.688, *n* = 18, and *P* = 0.0016), and values of MEM2 were significantly correlated with the latitude (Pearson’s *r* = −0.823, *n* = 18, and *P* = 0.000027) and longitude (Pearson’s *r* = −0.598, *n* = 18, and *P* < 0.0088). The percentage explained by the environmental, spatial, and leaf property fractions was 5.9, 12.5, and 0%, respectively, and the shared fraction between environmental and spatial variables explained 5.9% of the variation in fungal assemblages, indicating the fraction explained by MAT was spatially structured. In total, therefore, 12.5% of the variation in fungal assemblages was explained, and the remaining 87.5% was unexplained.

### Factors Relating to the Bleached Leaf Area

The bleached area accounted for 5.9% to 36.7% of the total leaf area at 18 sites (Table 1). The bleached area was significantly and negatively correlated with latitude and longitude and significantly and positively correlated with MAT, MAP, and the relative frequencies of *Gymnopus* sp. OTU_03, *Xylaria cubensis* OTU_07, *Gymnopus* sp. OTU_15, and Lachnocladiaceae sp. OTU_18 (Table 3). In contrast, the bleached area was not significantly (*P* > 0.05) correlated with the sampling month, elevation, maximum snow depth, LMA, AUR content, or the relative frequencies of other major OTUs. Stepwise model selection showed that MAT was the only predictor variable for the bleached leaf area (AIC = 108.73).

**TABLE 3:** Results of generalized linear models (GLMs) for relationships between the bleached area and the geographic and climatic factors, leaf properties, and the relative frequency of major fungal OTUs.

	Coefficient	Deviance	*P*
Latitude	–2.12	1062	***
Longitude	–1.23	608	***
MAT	2.59	1083	***
MAP	0.01	391	*
OTU_03 (*Gymnopus* sp., Basidiomycota)	4.04	370	*
OTU_07 (*Xylaria cubensis*, Ascomycota)	11.20	293	*
OTU_15 (*Gymnopus* sp., Basidiomycota)	16.38	296	*
OTU_18 (Lachnocladiaceae sp., Basidiomycota)	30.15	472	**

Results of simple regression of GLMs are shown; ***P < 0.001, **P < 0.01, and *P < 0.05. Those parameters with significant relationship at 5% level are indicated; the relationships with sampling month, elevation, LMA, AUR content, and the relative frequency of other major OTUs were not significant at 5% level and hence were not indicated. Abbreviations are as in Table 1.

## Discussion

The taxa of ligninolytic fungi associated with *C. sieboldii* leaf litter in Japan have been reported as common ones in temperate and subtropical forests. Fungi in Xylariaceae were reported to be common components of fungal assemblages on leaf litter and frequently isolated from bleached portions of leaf litter (Osono, 2020). *Crinipellis, Gymnopus, Marasmiellus, Marasmius*, and *Mycena* in Basidiomycota are reported to be associated with the decomposition of lignin in leaf litter (Miyamoto et al., 2000; Valášková et al., 2007). The fungal isolates obtained from bleached portions of *C. sieboldii* leaf litter exhibited potential bleaching activity (Table 2), indicating that they are responsible for the bleaching in the field. In fact, these basidiomycetous taxa have repeatedly been shown to be capable of selective removal of lignin and other recalcitrant compounds from leaf litter (Osono, 2020). In contrast, fungi in Xylariaceae have been demonstrated to have a degree of ligninolytic activity but generally cause selective decomposition of components other than lignin, in accord with previous findings that xylariaceous fungi prefer cellulose to lignin (Nilsson and Daniel, 1989; Pointing et al., 2003).

The composition of fungal assemblages showed a geographical pattern across 18 sites that was correlated with the latitudinal and longitudinal gradient of MAT, as shown by NMDS (Figure 2), PERMANOVA, and variation partitioning (Figure 3). In the variation partitioning, the MAT had a spatial structure, as these strongly correlated for the 18 study sites (Pearson’s *r* = 0.945, *P* < 0.001); hence, the effect of the latitude and MAT cannot be separated. Still, we detected significant effect of pure spatial fraction on fungal assemblages that was not shared by climatic variables (Figure 3), suggesting roles of unmeasured environmental variables that are spatially structured (Borcard and Legendre, 1994) or non-niche processes (Peay et al., 2016). In fact, some major OTUs were locally abundant and restricted to a few neighbor sites. For example, 12 (60%) of 20 isolates of *N. diffusa* OTU_02 were obtained from Site 10 (Hachijo Is.), and seven (64%) of 11 isolates of *Nodulisporium* sp. OTU_04 were from Site 1 (Iriomote Is.). The occurrence of locally-abundant OTUs implies a possible role of such non-niche processes as priority effect and dispersal limitation (Peay et al., 2010). In summary, the present study demonstrates the roles of climatic and spatial factors shaping the geographical pattern of ligninolytic fungi associated with the decomposition of leaf litter of single tree species.

The present study showed a clear gradient of the bleached area on the leaf surfaces of *C. sieboldii* which was greater at sites with higher MAT and precipitation located at lower latitudes and longitudes and at sites where *Gymnopus* spp., *Xylaria cubensis*, and Lachnocladiaceae sp. occurred at greater relative frequencies (Table 3). Previous studies have demonstrated that ligninolytic activity of fungi enhanced at higher temperatures (Osono, 2020) and at higher moisture condition (Fukasawa et al., 2013). Moreover, *Gymnopus* spp. and Lachnocladiaceae are shown to have potential capabilities to cause selective delignification in leaf litter (Osono et al., 2008). A few isolates of *X. cubensis* are reported to decompose lignin and other recalcitrant components actively (Osono, 2020), despite the general notion that xylariaceous fungi prefer cellulose to lignin, as discussed above. The result of the present study is consistent with the finding of a decomposition study showing that the decomposition of lignin and recalcitrant compounds is faster at warmer climates in an Asian climatic gradient (Osono, 2017). The present study suggests that not only the geographic location and climatic conditions but also the biogeographic patterns of distribution of ligninolytic fungi influence the decomposition of lignin in leaf litter.

The present study elucidated the biogeographic pattern of ligninolytic fungi associated with the bleaching of leaf litter of single tree species and the importance of climatic and spatial factors affecting the assembly of fungal assemblages. However, the explanatory power of climatic and spatial factors in the variation partitioning was low, and much of the variation in fungal assemblages remain unexplained. This suggests the importance of factors yet to be measured but affect the biogeography of bleaching fungi. Several factors may be invoked to explain such unexplained variation, such as tree species richness and composition and edaphic factors including nutrient levels (Zhang et al., 2018). Further studies are needed by incorporating more environmental variables to determine the main drivers of community assembly of ligninolytic fungi and to disentangle the relative importance of climatic, spatial, and other factors on their diversity and composition. We should note a further limitation of the present study that we used direct-observation and culture-dependent methods, which merits in confirming the potential bleaching activity for individual fungal isolates but could result in under-estimation of fungal richness due to selective retrieval of fungal isolates. Culture-independent methods such as DNA metabarcoding will be promising for comprehensive evaluation of the diversity and biogeography of ligninolytic fungi associated with the bleaching of leaf litter.

## Data Availability Statement

The datasets presented in this study can be found in online repositories. The names of the repository/repositories and accession number(s) can be found in the article/ Supplementary Material.

## Author Contributions

TO designed this study. TO, SM, and DH collected samples and data in the field. TO and DH analyzed the samples collected. TO and SM analyzed data. TO wrote the manuscript text and prepared figures and tables. All authors reviewed the manuscript.

## Conflict of Interest

The authors declare that the research was conducted in the absence of any commercial or financial relationships that could be construed as a potential conflict of interest.
